# Is low-dose amitriptyline effective in the management of chronic low back pain? Study protocol for a randomised controlled trial

**DOI:** 10.1186/s13063-016-1637-1

**Published:** 2016-10-22

**Authors:** Donna M. Urquhart, Anita E. Wluka, Malcolm R. Sim, Maurits van Tulder, Andrew Forbes, Stephen J. Gibson, Carolyn Arnold, Chris Fong, Shane N. Anthony, Flavia M. Cicuttini

**Affiliations:** 1 Department of Epidemiology and Preventive Medicine, School of Public Health and Preventive Medicine, Monash University, Alfred Hospital, Melbourne, VIC 3004 Australia; 2 Department of Health Sciences and EMGO+, Institute for Health and Care Research, VU University, 1081 HV Amsterdam, The Netherlands; 3 National Ageing Research Institute, Parkville, Melbourne, 3052 VIC Australia; 4 Caulfield Pain Management and Research Centre, Caulfield, Melbourne, 3162 VIC Australia; 5 Department of Anaesthesia and Perioperative Medicine, Monash University, Alfred Hospital, Melbourne, 3004 VIC Australia; 6 Eastern Health Clinical School, Faculty of Medicine, Nursing and Health Sciences, Monash University, Box Hill, Melbourne, 3128 VIC Australia; 7 Department of Medicine, Faculty of Medicine, Nursing and Health Sciences, Monash University, Melbourne, 3800 VIC Australia

**Keywords:** Antidepressants, Low back pain, Pain, Randomised controlled trial, Amitriptyline

## Abstract

**Background:**

Low back pain is a major clinical and public health problem, with limited evidence-based treatments. Low-dose antidepressants are commonly used to treat pain in chronic low back pain. However, their efficacy is unproven. The aim of this pragmatic, double-blind, randomised, placebo-controlled trial is to determine whether low-dose amitriptyline (an antidepressant) is more effective than placebo in reducing pain in individuals with chronic low back pain.

**Methods/design:**

One hundred and fifty individuals with chronic low back pain will be recruited through hospital and private medical and allied health clinics, advertising in local media and posting of flyers in community locations. They will be randomly allocated to receive either low-dose amitriptyline (25 mg) or an active placebo (benztropine mesylate, 1 mg) for 6 months. The primary outcome measure of pain intensity will be assessed at baseline, 3 and 6 months using validated questionnaires. Secondary measures of self-reported low back disability, work absence and hindrance in the performance of paid/unpaid work will also be examined. Intention-to-treat analyses will be performed.

**Discussion:**

This pragmatic, double-blind, randomised, placebo-controlled trial will provide evidence regarding the effectiveness of low-dose antidepressants compared with placebo in reducing pain, disability, work absenteeism and hindrance in work performance in individuals with chronic low back pain. This trial has major public health and clinical importance as it has the potential to provide an effective approach to the management of chronic low back pain.

**Trial registration:**

Australian New Zealand Clinical Trials Registry: ACTRN12612000131853; registered on 30 January 2012.

**Electronic supplementary material:**

The online version of this article (doi:10.1186/s13063-016-1637-1) contains supplementary material, which is available to authorized users.

## Background

Low back pain is a major public health problem worldwide. It is one of the most widespread health conditions, with a lifetime prevalence of more than 70 % in industrialised countries [1]. It is also the leading cause of disability globally, with estimates indicating that it is responsible for 83 million years lived with disability [2]. Moreover, low back pain is associated with substantial socioeconomic burden due to disability and productivity losses, with annual costs of AU$9.17 billion in Australia [3] and US$91 billion in the United States [4]. Although low back pain results in huge personal and socioeconomic costs, effective evidence-based treatments are limited.

Drug therapy is one of the treatment options available for chronic low back pain. Antidepressants are commonly prescribed in low back pain to treat depression, sleep and pain and their use is increasing for the treatment of low back pain [5]. Two main groups of antidepressants are used in the treatment of low back pain, tricyclic antidepressants (TCAs) and selective serotonin reuptake (SSRIs) inhibitors. While the mechanism of action of these antidepressants is not fully understood, their effect is attributed to inhibition of the reuptake of serotonin and/or noradrenaline in the central nervous system [6]. However, there is evidence to indicate that TCAs and SSRIs have an effect on the endogenous opioid system and can also act peripherally [7]. These mechanisms are thought to be important in targeting central sensitisation in chronic low back pain, where an abnormal state of responsiveness or increased gain in the nociceptive system occurs and neurons activated by nociceptive stimulus are sensitised and become hyperresponsive to subsequent stimuli [8]. Thus, antidepressants, at significantly lower doses (25–50 mg) than those which are used for depression (100–300 mg) are used in clinical practice to treat pain [6, 9]. In addition, the analgesic effect of low-dose antidepressants has been shown to be independent of their mood-altering effect [10].

Several systematic reviews have examined the efficacy of antidepressants in the management of chronic low back pain [11–13]. However, these reviews have reached different conclusions. A Cochrane systematic review and meta-analysis, which evaluated the effectiveness of TCAs (seven studies), SSRIs (three studies) and ‘atypical’ antidepressants (two studies), concluded that there is no clear evidence to indicate that antidepressants are more effective than placebo in reducing pain in individuals with chronic low back pain [14]. Moreover, pooled analyses showed no difference in pain relief between different types of antidepressants and placebo. However, a high-quality trial by Atkinson [15], which performed a head-to-head comparison of TCAs and SSRIs, reported a greater reduction in pain intensity with a low concentration of a TCA than with all concentrations of a SSRI. The authors concluded that the Cochrane review was limited by a paucity of trials, in particular those examining low-dose antidepressants, small study populations and variation in study quality and individuals recruited. Further trials were recommended to confirm the efficacy of antidepressant therapy for chronic low back pain. Thus, the role of antidepressants in low back pain is unclear and although low-dose antidepressants are a common treatment for chronic low back pain, their use is still unproven for the treatment of pain.

We propose a pragmatic, double-blind, randomised, placebo-controlled trial to determine whether a low-dose TCA (amitriptyline) is effective in reducing pain. It is hypothesised that low-dose amitriptyline will improve pain in individuals with chronic low back pain. If it is found to be effective, it will provide high-quality evidence for its use and potentially enable this treatment option to be considered by more individuals with chronic low back pain. If we do not find low-dose amitriptyline to be effective, then our trial will provide strong evidence to reconsider its use in the management of chronic low back pain.

## Methods

### Study design

This study is a double-blind, randomised, placebo-controlled trial, with a two-arm, parallel group, superiority design. The trial was registered at the Australian New Zealand Clinical Trials Registry prior to recruitment (ACTRN: ACTRN12612000131853) and trial reporting will be guided by the Consolidated Standards of Reporting Trials (CONSORT) [16] and Standard Protocol Items: Recommendations for Interventional Trials (SPIRIT) guidelines (See Additional file 1). Ethics approval has been obtained from the Alfred Hospital Ethics Committee (HREC/12/Alfred/16, 476/11), Monash University Human Research Ethics Committee (CF12/0271 - 2012000106) and the Eastern Health Human Ethics Committee (SERP28/1112).

### Participants

A total of 150 individuals with chronic low back pain will be recruited through hospital and private medical and allied health clinics, advertising in local media including national newspapers and community magazines, and posting of flyers in community locations such as shops, libraries and medical clinics. Written informed consent will be obtained from all participants by research staff trained in study procedures specific to this trial.

### Inclusion criteria

We will recruit male and female participants aged 18–75 years with chronic, nonspecific low back pain, which is defined as pain between the lower borders of the rib cage and the gluteal folds, is without a specific cause and has been present for longer than 3 months [17, 18].

### Exclusion criteria

Participants with any of the following will be excluded: (1) specific pathological entity, such as infection, metastasis, osteoporosis or fractures, (2) major coexisting illness which might confound assessment of function or for which amitriptyline may be inappropriate (e.g. heart or thyroid problems, glaucoma, seizures, urinary issues), (3) another significant musculoskeletal condition, (4) history of psychosis, (5) current or previously diagnosed depression with or without the use of medication), (6) any prior or current use of antidepressants, (7) current use of opioids, (8) any contraindication or allergy to amitriptyline, (9) pregnancy, planning or trying to become pregnant or breastfeeding or (10) inability to give informed consent, including individuals that are unable to read, speak or understand English.

### Randomisation and blinding

Randomisation will be based on computer-generated random numbers prepared by a statistician who will have no involvement in the trial. The allocation of participants will be in a 1:1 ratio to either the intervention or the control group. The use of a central allocation which involves pharmacy-controlled randomisation will ensure that the allocation cannot be accessed or influenced by any of the research personnel. The randomised controlled trial (RCT) will be double-blinded, with both participants and investigators assessing outcomes blinded to treatment allocation. Allocation concealment and double blinding will be ensured by (1) the medications being dispensed by the hospital clinical trial pharmacy, (2) the use of an identical active placebo tablet which mimics the side-effects of amitriptyline and (3) questionnaire data that is subjectively being taken by research assistants blinded to group allocation.

### Intervention

Participants in the intervention arm will receive a TCA, 25 mg of amitriptyline (Alphapharm Pty Ltd., Miller’s Point, NSW, Australia), and those in the control arm will receive an active placebo, 1 mg benztropine mesylate (Phebra Pty Ltd., NSW, Australia). Amitriptyline and benztropine will be administered in identical capsules to be taken in a single dose at the same time each day. We selected low-dose amitriptyline as it is commonly prescribed for the management of chronic pain [19]. It has been reported to be the most effective antidepressant for the treatment of neuropathic pain, such as diabetic neuropathy and neuralgia [20], and is also effective for various pain conditions, including fibromyalgia [7], ankylosing spondylitis [21] and headaches [22]. Amitriptyline can act on pain independent of depression [10], and while side effects, such as dry mouth, mild constipation and fatigue, can occur, lower doses are used for pain modification and are generally well-tolerated. We have selected benztropine, an active placebo, as it mimics the side effects of amitriptyline, such as dry mouth and constipation, while having no known effect on chronic pain [23, 24]. All participants will be provided with usual care by their treating health practitioners.

### Study procedure

**Fig. 1:**
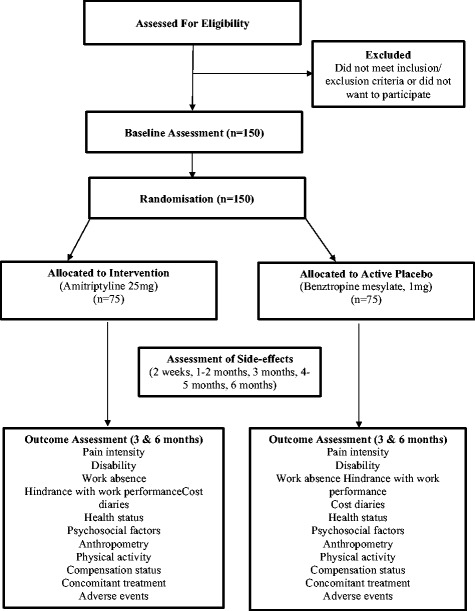
Trial flow diagram

The study procedures are presented in Fig. 1. Potential participants will be initially screened over the phone using a questionnaire to determine whether they meet the eligibility criteria. They will then attend an initial assessment at Monash University Department of Epidemiology and Preventive Medicine with the aim of obtaining informed consent and confirming the individual’s eligibility to participate in the trial. Eligible participants will be randomised, complete a baseline assessment and receive the first 3 months of amitriptyline or benztropine from the Alfred Hospital Clinical Trials Pharmacy. Participants will be contacted by phone at 2 weeks, 1–2 months, 3 months, 4–5 months and 6 months to monitor their progress and any side effects of the treatment. The 3- and 6-month outcome questionnaires and the second 3 months of medication will be posted to the participants by mail. The same researchers, who are blinded to treatment allocation, will measure all clinical variables, administer questionnaires, monitor compliance and record adverse events. Unblinding will be allowed under certain circumstances, such as a participant’s physician requiring their allocated intervention to ensure that they receive the appropriate medical care. Compliance by trial medication will be assessed by pill count. Participants will not be paid for their participation in the trial, but they will be reimbursed for parking and transport costs.

### Outcome measures

The following primary and secondary, self-report outcome measures will be administered by blinded research assistants at baseline and 3 and 6 months.

#### Primary outcome measures: pain intensity

Our primary outcome measure will be pain intensity measured at 6 months using a Visual Analogue Scale (VAS) of 100 mm. However, the Descriptor Differential Scale, a valid measure of pain intensity, will also be assessed [25]. The Descriptor Differential Scale has been used in a previous trial of antidepressants in low back pain [15], enabling calculation of study sample size and comparison of results.

#### Secondary outcome measures: disability, work absenteeism and hindrance in work performance

Disability will be assessed using the Roland Morris Disability Questionnaire [26], which is a validated instrument designed to assess self-rated disability caused by low back pain.

We will examine absenteeism and hindrance in performance of paid and unpaid work using The Short Form Health and Labour questionnaire (SFHLQ). The SFHLQ is a validated questionnaire which examines work absenteeism and hindrance in performance of paid and unpaid work in relation to injury or sickness [27].

### Other measures

Global improvement: a 6-point scale, ranging from 'no change' to a 'great deal better' will be used to assess global improvement [28].

General health status will be measured using the EuroQol, 5 dimensions instrument (EQ-5D-5 L) [29].

Depression: severity of mood symptoms will be assessed using the Beck Depression Inventory [30].

Fear of movement/(re)injury will be examined using the 17-item Tampa Scale [31].

### Potential explanatory factors

#### Anthropometry

Height (stadiometer), weight (electric scales) and Body Mass Index (height/weight^2^) will be measured at baseline.

#### Physical activity

The International Physical Activity Questionnaire short version [32] will be used at baseline, and 3 and 6 months.

#### Concomitant medication use

The use of non-opioid analgesics and non-steroidal anti-inflammatory drugs (NSAIDs) will be allowed during the trial. The randomisation process is the most effective method for ensuring that the two groups are as similar as possible with respect to known confounders and unknown potential confounders including treatments. We will also adjust for medication use in the analyses.

#### Compensation status

For each individual we will record whether their back pain is associated with a compensation claim, and if so the nature of the claim, including the type, duration, items approved and associated costs.

#### Monitoring

Adverse events will be monitored on a monthly basis over the phone using a validated questionnaire, the UKU Side Effects Rating Scale [33]. This questionnaire involves a standardised, physician-administered interview for assessing the severity and impact of side effects of psychotropic drugs on daily function. The participants will be requested to report any adverse events to the research staff spontaneously. Details of major adverse events and their relationship with study intervention will be recorded and reported to the Ethics Committees. The participants’ physician will also be notified of their inclusion in the trial.

### Sample size calculation

#### Reduction in pain intensity

A previous clinical trial of TCAs in individuals with chronic low back pain reported the mean (standard deviation (SD)) pain scores measured on the VAS to reduce to 5.70 (2.43) in the placebo group (*n* = 48) [34]. With *n* = 56 in each arm of the study, we have 90 % power to detect a difference of 1.5 (i.e. VAS to reduce to 4.20 (2.43) in the treatment group), which reflects the minimum clinically significant reduction in pain intensity (*α* = 0.05, two-sided significance).

In a recent study of individuals with low back pain, the control group (*n* = 22) had a pain score that reduced to a mean (SD) = 6.2 (2.8) on the Descriptor Differential Scale following treatment, while the pain score of the treatment group (TCA) (*n* = 19) reduced to a mean (SD) score = 4.5 (2.6) [15]. With an *α* = 0.05 and *n* = 60 in each arm of the study, we have 90 % power to detect a difference of this size (1.7), which reflects clinically significant reductions in pain intensity from ‘moderate’ to ‘mild’ or from ‘strong’ to ‘moderate’, which is equivalent to a decrease of 2 units (17 %) on the Descriptor Differential Scale (0–12 per descriptor) [35].

#### Improvement in disability

With 60 in each arm of the study, we will have 90 % power to detect a clinically relevant difference in disability (improvement of 13–16 % in disability or 3–4 points on the 24-point Roland Morris Disability Questionnaire after 26 weeks [36]) (*α* = 0.05, two-sided significance), which corresponds to a significant improvement in key functional activities, including walking and dressing.

More generally, with 60 per arm we have 90 % power to detect a difference of 0.60 SDs. With our primarily analyses involving adjustment for the baseline value of the outcome, we will have greater than 90 % power according to the size of the baseline-follow-up correlation. Given our previous experience in such studies we expect a maximum dropout rate of 20 % so we will recruit a total of 150 (75 in each arm of the study) [37].

### Statistical analysis

Summary statistics comparing randomised arms at baseline will be tabulated. Intention-to-treat analyses of primary and secondary continuous outcomes will be performed by linear regression adjusting for the baseline of the outcome variable where relevant. Logistic regression will be performed for binary outcomes. Adjustment for imbalanced baseline factors, including the presence of symptoms other than low back pain, will be performed as supplementary analyses. Analyses of treatment efficacy will be done by censoring individuals at the time of any protocol deviation and developing a model for the probability of deviation, followed by weighted analyses using only the uncensored individuals where the weights are the inverse probability of censoring. This produces estimates of treatment effect as if there was full compliance with the protocol in this RCT and is far preferable to per-protocol analyses based on (unweighted) observed compliance [38]. This RCT is well-placed to model noncompliance with frequent monitoring for adverse events and resultant prognostic information.

### Data integrity and management

All collected data will be recorded using Case Report Forms or questionnaires and stored in a locked area in the Department of Epidemiology and Preventive Medicine at Monash University with secured and restricted access. The electronic data will be stored in a password-protected database with secured and restricted access. All data collected will be kept strictly confidential. Data transfer will be encrypted with all data deidentified. Only research personnel on the project will have access to the study data.

### Withdrawal

If participants withdraw before completion of the study, the reason and date will be recorded and participants will be asked if they can complete the remaining outcome measures.

### Monitoring

The principal investigators will monitor the conduct and progress of the project and ensure that all trial procedures are compliant with the trial protocol. The research team will have regular meetings to ensure efficient study execution and ongoing monitoring of adverse events.

## Discussion

We have proposed to conduct a pragmatic, double-blind, randomised, placebo-controlled trial to investigate whether low-dose amitriptyline is more effective than placebo in the management of pain in individuals with chronic low back pain. We will also investigate whether lose-dose amitriptyline is more effective than placebo in improving disability and minimising absenteeism and hindrance with performance of paid/unpaid work. If amitriptyline is found to be effective, it will provide high-quality evidence for this therapeutic approach to the management of chronic low back pain.

This current trial of amitriptyline for chronic low back pain was designed to address some of the limitations of previous RCTs which have been identified in meta-analyses. The most up-to-date meta-analyses by the Cochrane collaboration reported that a qualitative analysis of the trials with a low risk of bias found conflicting evidence regarding the effectiveness of antidepressants in the reduction of pain for individuals with chronic low-back pain, while a meta-analysis of six trials showed no difference in pain relief between antidepressants and placebo [14]. The review highlighted a number of limitations including a paucity of trials that examined antidepressants for the treatment of pain, in particular those which used a low-dose antidepressant. Moreover, small sample sizes, which can result in insufficient statistical power to detect clinically relevant effects, was a key limitation. Of the six trials identified in the Cochrane meta-analysis examining the effect of low-and high-dose antidepressants on pain, study populations ranged from 18 to 92 (randomised) subjects. In this current clinical trial we aim to recruit 150 subjects with chronic low back pain. If successful, it will be the largest trial of an antidepressant for the treatment of pain in participants with low back pain to date. A further methodological issue that was reported was the short follow-up periods of the trials [14]. These periods were generally between 6 and 8 weeks; however, one study followed up participants only until 4 weeks [39], while a single study extended to 12 weeks [15]. We have selected to follow-up participants over 6 months to enable the efficacy of the low-dose antidepressants to be examined over a longer time period.

Previous trials that have examined the efficacy of antidepressants for treating pain in low back pain have varied in both the percentage of participants recruited with and without depression and the severity of depression of the included participants. While one study specifically selected participants with overt symptoms of depression [34] and two studies have included a mix of participants with and without depression [39, 40], two studies excluded individuals with major depression [15, 41] and, in a further study, it was unclear whether participants had depression [42]. We have chosen to exclude potential participants with depression or psychosis to allow us to examine the effectiveness of low-dose amitriptyline specifically on pain. Finally, we have chosen to use an active placebo, benztropine mesylate. Only two of the previous studies used an active placebo, with the remaining studies selecting a standard, inactive placebo. The benefit of using benztropine mesylate is that it mimics the side-effects of amitriptyline and assists with blinding of participants.

This trial has several potential limitations that need to be considered. Given that we are examining the effectiveness of low-dose amitriptyline, which is a TCA, it is not possible to directly extrapolate the findings to higher doses of TCAs or other types of antidepressants. While a variety of different types of antidepressants have been examined in the management of low back pain, much of the investigation has focused on TCAs and SSRIs. TCAs and SSRIs block the absorption of the chemicals or neurotransmitters, serotonin and/or noradrenaline, at the spine and midbrain [6]. There is preliminary evidence to suggest that low-dose TCAs produce greater reduction in pain compared with SSRIs and placebo. For instance, a study by Atkinson [15], which performed a head-to-head comparison for TCAs and SSRIs, showed an effect size of 0.31 (−0.23, 0.87) for fluoxetine (SSRI) and −0.62 (1.24, −0.01) for desipramine (TCA). This provides preliminary evidence suggesting that low-dose TCAs produce greater reduction in pain compared with placebo and SSRIs. However, this is still not proven in the treatment of low back pain as meta-analyses show no significant differences in pain between the TCA and SSRI treatment groups [14].

The current trial will involve the recruitment of participants with chronic, nonspecific low back pain. While this means that our population sample has the potential to be heterogeneous in nature, the participants recruited will represent a distinct clinical entity; that is they will have pain that has been present for longer than 3 months, their pain will be of no known cause and participants will have no diagnosed psychosis or depression. A further limitation of this trial relates to examining the adverse events associated with the use of low-dose antidepressants. While in our evaluation of the effectiveness of amitriptyline we will record the type, frequency and severity of adverse events that occur during the trial, the study is not designed to evaluate adverse events and prospective cohort studies with larger sample sizes are needed to examine the incidence of adverse events.

In summary, low back pain is the leading cause of disability worldwide, but effective treatment approaches are limited. Low-dose antidepressants are commonly used in clinical practice to treat pain in individuals with chronic low back pain; however, their use is unproven. This RCT will provide high-quality evidence to investigate whether low-dose amitriptyline is an effective treatment in the management of low back pain. If amitriptyline is found to be effective, it could be used to reduce pain, disability and work absenteeism and hindrance of work performance in individuals with chronic low back pain, and in turn, reduce the need for treatments and surgery that result in substantial costs to the community.

## Additional file

Additional file 1: SPIRIT 2013 checklist: Recommended items to address in a clinical trial protocol and related documents. (DOC 121 kb)

## Abbreviations

RCT: Randomised controlled trial
SSRI: Selective serotonin reuptake inhibitor
TCA: Tricyclic antidepressant
